# Successful delivery after in vitro fertilization-embryo transfer in a woman with metachronous primary cancer of ovary and endometrium: a case report

**DOI:** 10.1186/s12884-023-05973-z

**Published:** 2023-09-19

**Authors:** Yichang Tian, Yu Liang, Xiaokui Yang

**Affiliations:** 1 Department of Human Reproductive Medicine, Beijing Obstetrics and Gynecology Hospital, Capital Medical University, Beijing, China; 2 Beijing Maternal and Child Health Care Hospital, Beijing, China

**Keywords:** Endometrial cancer, Intraepithelial carcinoma, Placenta previa, Placenta accreta spectrum, In vitro fertilization-embryo transfer (IVF-ET), Case report

## Abstract

**Background:**

The appearance of malignancies at various times in the same individual, excluding metastases of the initial primary cancer, is termed multiple primary cancers. Double primary gynecological cancers cause inevitable damage to female reproductive function, and the preservation of fertility in such patients remains a challenging issue as relatively few cases have been reported. This case report provides management options for dual primary ovarian and endometrial cancers, including the choice of ovulation induction protocols, considerations during pregnancy and parturition, with the aim of providing assistance to clinicians.

**Case presentation:**

We report a case of a 39-year-old woman with primary infertility and a medical history of right-sided ovarian mucinous borderline tumor with intraepithelial carcinoma, left-sided ovarian mucinous cystadenoma and endometrial cancer, who successfully conceived with in vitro fertilization-embryo transfer (IVF-ET) after three different ovulation induction protocols. During her pregnancy, she was complicated by central placenta praevia with placental implantation and eventually delivered a healthy female infant by caesarean section at 33 gestational weeks.

**Conclusions:**

For patients with double primary gynecological cancers who have an intense desire for fertility, the most appropriate oncological treatment should be applied according to the patient’s individual situation, and fertility preservation should be performed promptly. Ovulation induction protocol should be individualized and deliberate, with the aim of ensuring that the patient’s hormone levels do not precipitate a recurrence of the primary disease during induction of ovulation and maximizing fertility outcomes. In addition, the risk of postpartum hemorrhage due to placental factors cannot be neglected in such patients.

## Background

Considerable advances have been achieved in the diagnosis of malignant tumors and an increasing number of patients are being diagnosed at an earlier stage of cancer [1]. As a consequence, the incidence of cancer continues to rise year on year, with statistics estimating that there are approximately 19.3 million new cases worldwide in 2020 and this figure will reach a staggering 28.4 million in 2040 [2]. In parallel, a growing number of individuals are suffering from multiple types of cancer throughout their lives [3]. Multiple primary cancers are defined as two or more malignancies occurring in the same individual, and the subsequent cancer cannot be metastases of the initial primary tumor. Based on the Surveillance, Epidemiology, and End Results (SEER) Program recommendations, multiple primary cancers can be further subdivided into synchronous or metachronous depending on whether the time interval between initial and secondary diagnosis is greater than two months [4]. The prevalence of multiple primary cancers has been on the rise recently and it should not be overlooked that females are more likely to experience multiple primary cancers in comparison to males [5]. Among women with multiple primary cancers, the most noteworthy type of cancer is undoubtedly gynecological malignancy, since the management of this type of tumor will inevitably result in the removal or damage of female reproductive organs, thus affecting their fertility. Thus, how to preserve the fertility of patients with multiple primary gynecological cancers while treating them is an emerging and unavoidable issue in the area of reproductive medicine. Assisted reproductive technology (ART) is an important modality for fertility preservation in patients with malignancy, but due to the higher risk of recurrence resulting from fertility-sparing surgery (FSS), in vitro fertilization-embryo transfer (IVF-ET) is generally preferred over natural conception after FSS.

**Fig. 1:**
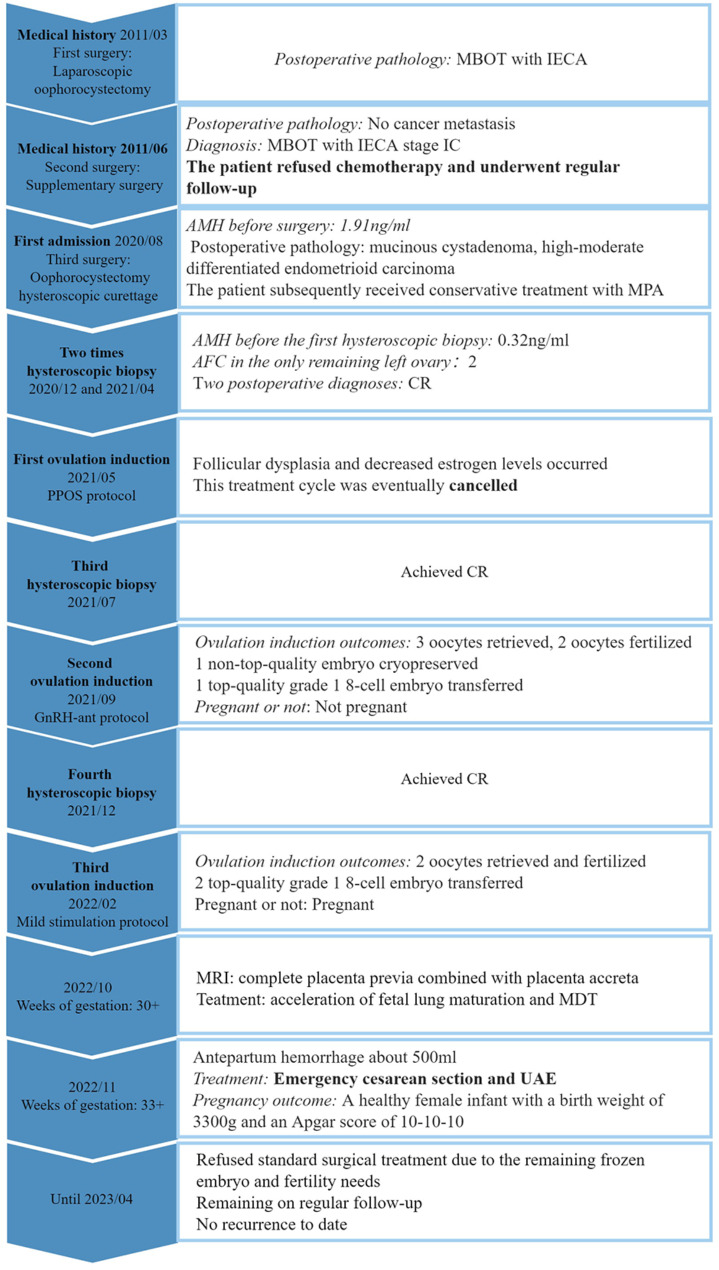
Timeline of the entire case treatment procedure. Abbreviations: MBOT, Mucinous borderline ovarian tumors; IECA, Intraepithelial carcinoma; AMH, Anti-Müllerian hormone; MPA, Medroxyprogesterone acetate; AFC, Antral follicle count; CR, Complete response; PPOS, Progestin-primed ovarian stimulation; GnRH-ant, Gonadotropin releasing hormone antagonist; MRI, Magnetic resonance imaging; MDT, Multi-disciplinary treatment; UAE, Uterine artery embolization

In this case report, we present a woman with a medical history of right-sided mucinous borderline ovarian tumor (MBOT) with intraepithelial carcinoma (IECA), left-sided ovarian mucinous cystadenoma, endometrial adenocarcinoma who underwent IVF-ET and conceived after receiving three different ovulation induction protocols, followed by complete placenta praevia combined with placental implantation during gestation, resulting in a successful delivery. As far as our knowledge extends, successful deliveries following IVF-ET treatment in such patients are extremely rare. We believe that this case is clinically instructive and that it has lessons and insights into the treatment of patients with multiple primary malignancies in terms of preserving fertility, choosing ovulation protocols and managing during pregnancy. The timeline of the entire treatment process is illustrated in Fig. 1.

## Case presentation

A 36-year-old nulligravida presented this medical center in August 2020 with an approximately 11 cm left-sided adnexal cystic mass and inhomogeneous endometrium under ultrasound scan.

The patient had previously undergone laparoscopic oophorocystectomy of right-sided ovarian cyst in another medical center in March 2011. Postoperative pathology diagnosed MBOT with IECA, thus supplementary treatment with transabdominal right-sided salpingectomy, omentum resection, appendectomy, pelvic lymph node dissection, peritoneal and omental biopsies was preformed three months later. The postoperative pathology did not indicate any evidence of cancer metastasis, thus confirming the tumor stage as IC. However, the patient refused chemotherapy due to her strong desire for fertility. Subsequently, she underwent regular follow-up every 3–6 months as required.

**Fig. 2:**
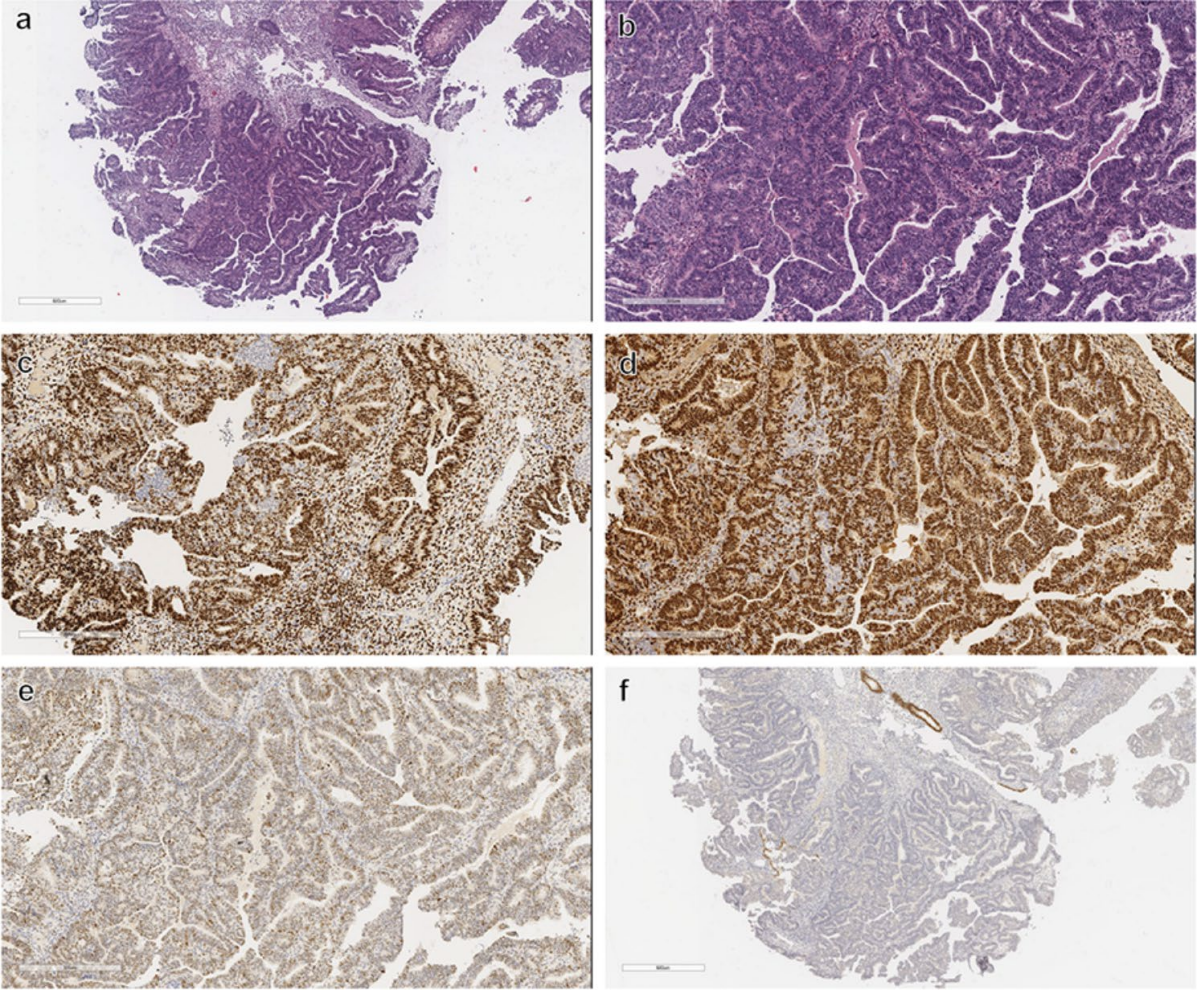
Postoperative histopathological and immunohistochemical results of the first hysteroscopic curettage. **a** H&E × 40. **b** H&E × 100. **c** positive expression of estrogen receptor. (Magnification, × 100.) **d** positive expression of progesterone receptor. (Magnification, × 100.) **e** wild-type pattern expression of p53. (Magnification, × 100.) **f** negative expression of PAX2. (Magnification, × 40.)

**Fig. 3:**
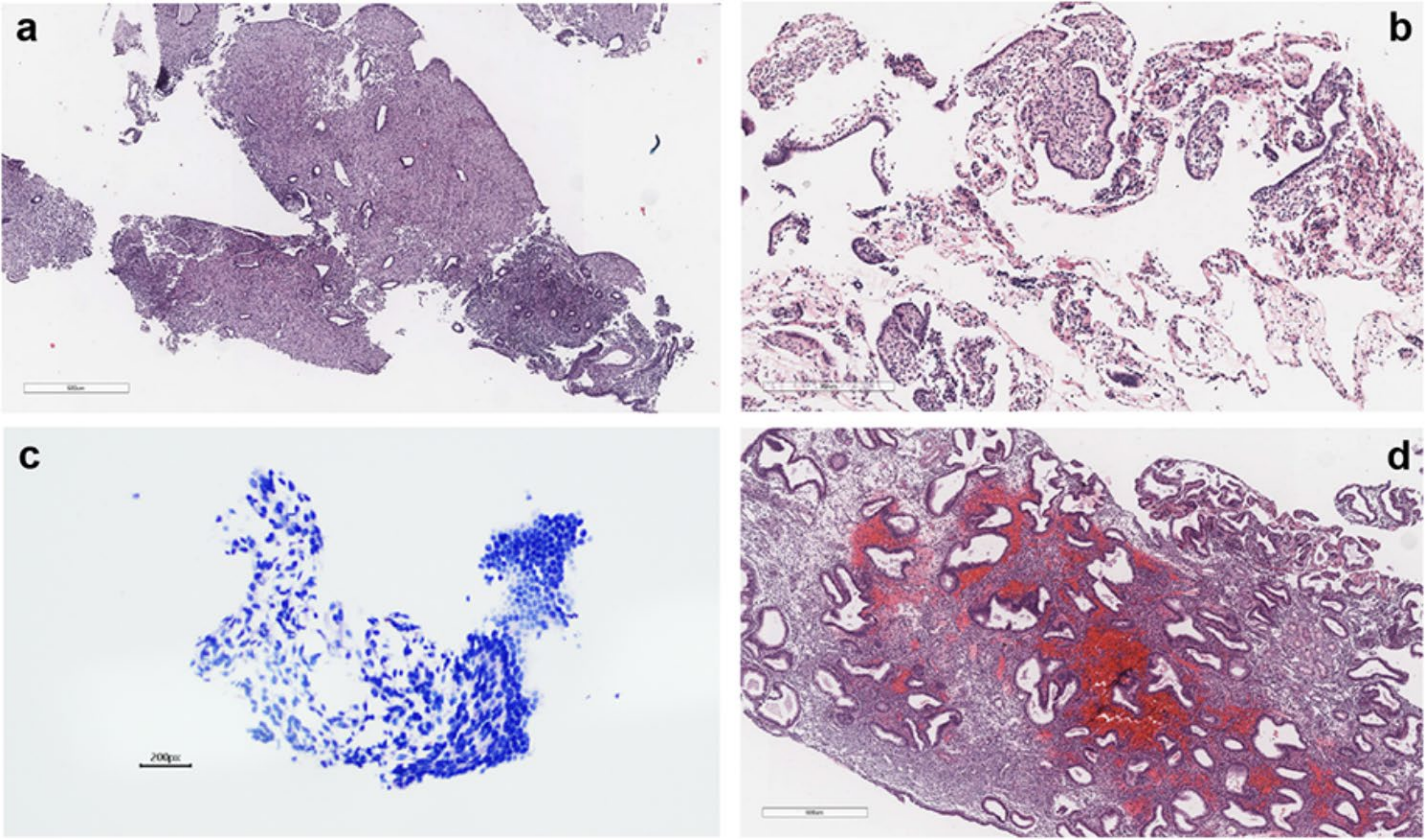
Postoperative pathological results of four hysteroscopic biopsies. **a** pathology result in December 2020 (H&E × 40.) **b** pathology result in April 2021 (H&E × 100.) **c** pathology result in July 2021 (H&E × 200.) **d** pathology result in December 2021 (H&E × 100.)

The patient completed the MRI after hospitalization in our medical center, which suggested a complex primarily cystic mass in the left upper side of the uterus, measuring approximately 8.9*10.2*9 cm; the endometrium was heterogeneous, and malignancy was not excluded due to the high DWI signal. Tumor marker results indicated no abnormalities. The patient’s preoperative assessment of ovarian reserve was within normal range (anti-Müllerian hormone, AMH: 1.91 ng/ml). Considering the her history of two pelvic surgeries thus a higher likelihood of pelvic adhesions and the relatively large size of the tumor, we performed a transabdominal procedure. Consequently, she underwent transabdominal left-sided oophorocystectomy and hysteroscopic curettage in August 2020. Postoperative pathological findings suggested mucinous cystadenoma of the left ovary and high-moderate differentiated endometrioid carcinoma (Fig. 2). The patient strongly requested to preserve her fertility, but on recheck her AMH was only 0.32 ng/ml and the preserved left ovary had only two antral follicles on ultrasound. With the patient’s full knowledge, she was treated conservatively with oral medroxyprogesterone acetate (MPA; Beijing Zhong Xin Pharmaceutical, China) 500 mg/day for 6 months postoperatively. Thereafter, she underwent two times of hysteroscopic endometrial pathological evaluations in December 2020 and April 2021 (Fig. 3a-b), while magnetic resonance imaging (MRI) was performed to clarify the absence of tumor presence in the thoracic, abdominal and pelvic cavities, and the patient was considered to have achieved complete response (CR).

The patient received her first cycle of ovulation induction via progestin-primed ovarian stimulation (PPOS) protocol on 10 May, 2021, menstruation cycle day (MC) 3, using a combination of human menopausal gonadotropin (hMG; Livzon Pharmaceutical Factory, China) at a dose of 150 IU/day and 10 mg/day oral MPA. Due to the poor ovarian response, the dose of hMG was increased to 225 IU/day on MC 7, the follicles were still observed to be atrophied and the level of estrogen was also decreased on MC 12, hence this treatment cycle was eventually terminated.

After a third hysteroscopic assessment in July 2021 to determine that she was remaining in CR (Fig. 3c), she received her second treatment cycle with gonadotropin releasing hormone (GnRH) antagonist regimen in September 2021. On MC 3, she was administered hMG 150 IU/day and GnRH antagonist (Cetrorelix; Merck-Serono, Spain) 0.25 mg/d was added on MC 8 depending on the ovarian response. She was triggered with 250 μg recombinant human chorionic gonadotropin (hCG; Ovidrel, Merck Serono Inc, Geneva, Switzerland) on MC 11 and three oocytes were retrieved 36 h later. Two oocytes were normally fertilized with her husband’s semen. On the third day after fertilization, one non-top-quality embryo was frozen by vitrification. The other top-quality grade 1 8-cell embryo was transferred in the same day, but eventually failed to conceive.

**Fig. 4:**
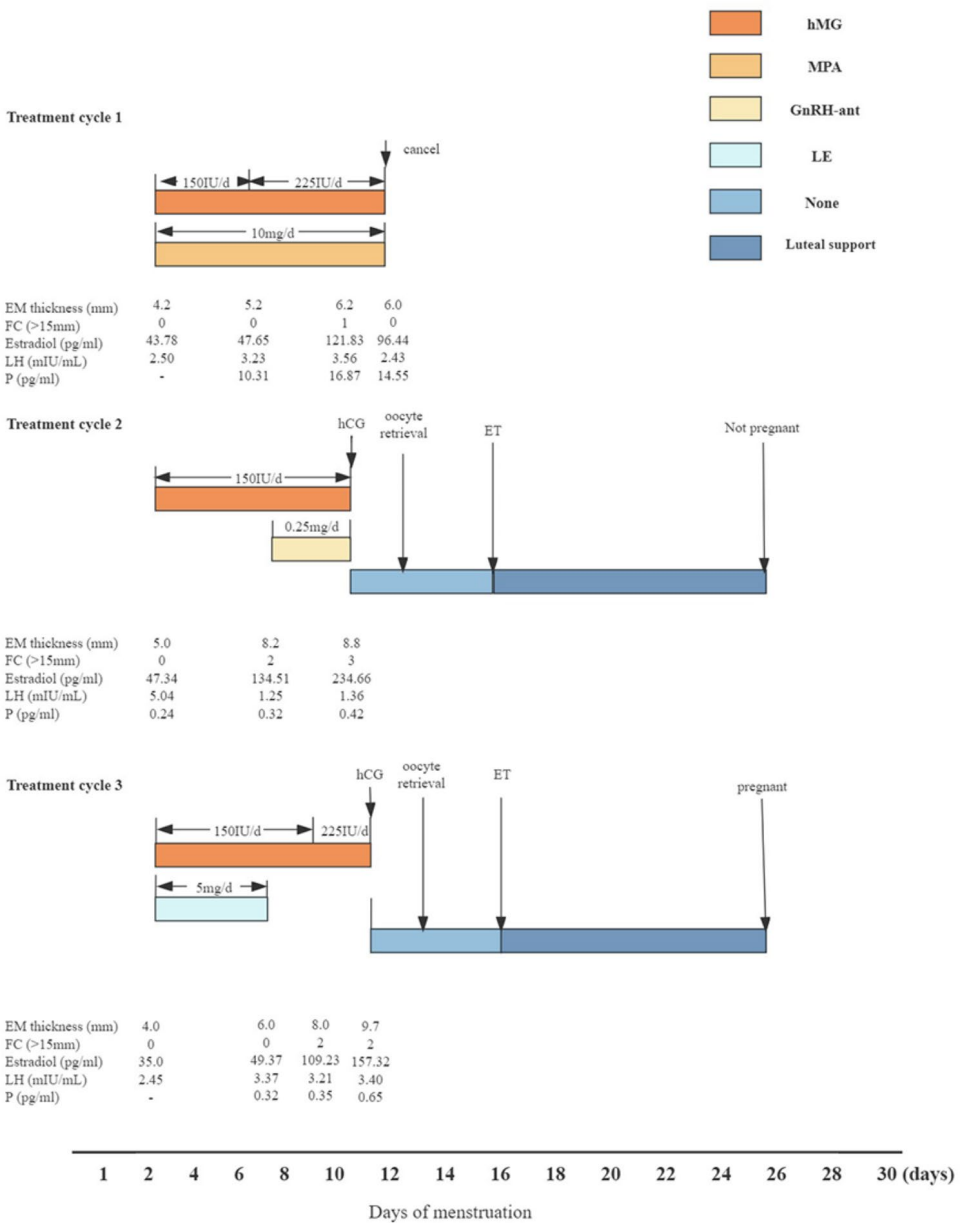
Detailed clinical course of three different ovarian stimulation treatment cycles. Abbreviations: EM, endometrium; FC, follicle count; LH, luteinizing hormone; P, Progesterone; hMG, human menopausal gonadotropin; hCG, human chorionic gonadotropin; IU, international unit; MPA, Medroxyprogesterone acetate; GnRH-ant, Gonadotropin releasing hormone antagonist; LE, Letrozole; ET, Embryo transfer

The patient underwent her fourth hysteroscopy in December 2021 and it was determined that she still achieved CR (Fig. 3d). Therefore, in February 2022, she began the third treatment cycle with the mild stimulation protocol for ovulation induction, using the combination of Letrozole (LE; Fu Rui, Jiangsu Hengrui Pharmaceutical Co., China) and hMG. She received LE 5 mg/day orally from MC 3 to 7. hMG with a dosage of 150 IU/day was applied from MC 3, adjusted to 225 IU/day on MC 10 continue with this dose until the day before the trigger with 250 μg hCG. Transvaginal oocyte retrieval was preformed 36 h later. Two oocytes were collected and both of them were fertilized. Three days after retrieval, two top-quality grade 1 8-cell embryos was transferred. She subsequently received luteal support and resulted in a positive pregnancy test 10 days later. Detailed clinical course of three ovarian stimulation treatment cycles was presented in Fig. 4.

**Fig. 5:**
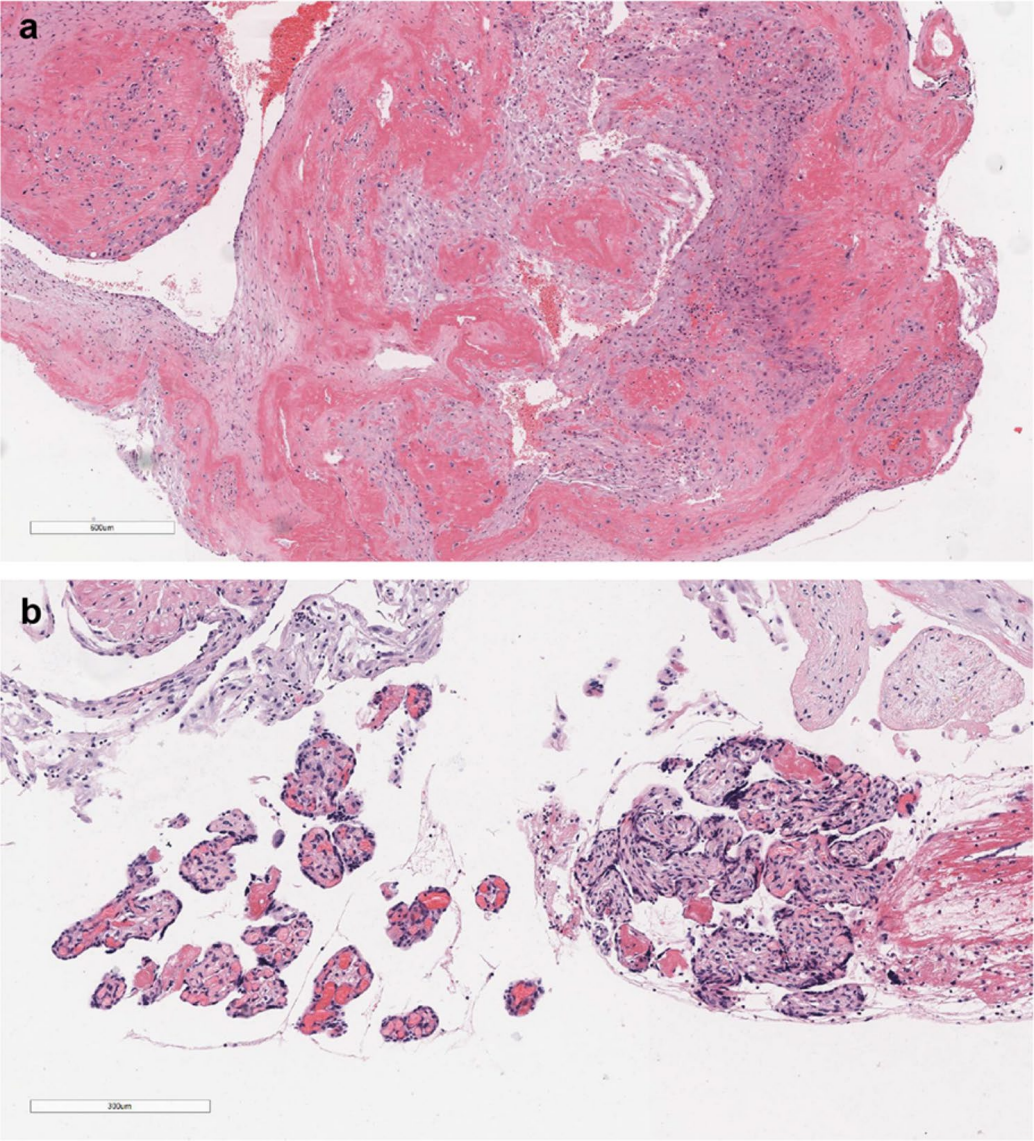
Postoperative pathological results of the caesarean section. **a** decidualized tissue (H&E × 40.) **b** placental villi tissue (H&E × 100.)

A clinical pregnancy was confirmed by visualization of a gestational sac on ultrasonographic examination 30 days after the embryo transfer, and thereafter regular pre-natal examinations were performed as scheduled. A placental MRI performed at 30 + weeks of gestation revealed complete placenta previa combined with placenta accreta, which was treated with acceleration of fetal lung maturation after a comprehensive assessment. Meanwhile, multi-disciplinary physicians including gynecologic oncology, critical care medicine, and neonatology were also invited for consultation to formulate further treatment plan for the patient. At 33+ weeks of gestation, the patient presented with antepartum hemorrhage with approximately 500ml, therefore an emergency lower uterine transverse incision cesarean section, ligation of the superior branch of the right uterine artery, endometrial biopsy was performed. Since intraoperative hemorrhage was about 1500 ml, the patient was given appropriate rehydration treatment and uterine cavity tamponade, and the multi-disciplinary treatment (MDT) was proceeded as planned. At the end of the procedure, minor hemorrhage was still noted by pressing on the uterine fundus, thus bilateral uterine artery embolization (UAE) was administered. The patient eventually delivered a healthy female infant, with a birth weight of 3300 g and an Apgar score of 10-10-10. No abnormalities were found in the postoperative pathology (Fig. 5).

The patient has not undergone standard surgical treatment as she still retains one frozen embryo and has fertility needs. She remains on regular follow-up and has not recurred to date.

## Discussion

With the increasing incidence of malignant tumors and the tendency of younger age at onset, patients with multiple primary neoplasms were no longer a novelty, since as early as 1921, it was reported that approximately 4.7% of 3000 cancer patients suffered from multiple primary malignancies [6]. In recent years, the actual incidence of multiple primary cancers demonstrates an upward trend due to the continuous advances in cancer diagnostic techniques and the evolution of epidemiological investigation approaches: while a literature review in 2003 indicated that the prevalence of multiple primary cancers ranged between 0.7% and 11.7% [7], this figure has leapt to between 2.4% and 17.2% in another comprehensive review in 2017 [1]. In the past, prolonging survival time was an indispensable measure of oncology treatment, but as therapeutic advances have led to a remarkable increase in cancer survival rates over the past 20 years, quality of life has gradually become an essential component in evaluating outcomes, and reproductive health is an integral part of quality of life. However, the harsh truth is that 80% of oncology patients are facing reduced fertility after anti-tumor treatment, particularly in patients with multiple primary cancers, thus preserving the fertility of such patients is a crucial matter [8].

MBOT with IECA, which was first proposed by Riopel et al. in 1999, is developed on the basis of MBOT and is a transitional stage in the process of ovarian carcinogenesis [9]. In recent years, MBOT with IECA have gradually gained attention, but due to the low incidence, the majority of clinical management of IECA follows the treatment of ovarian cancer (OC). Since patients with IECA are relatively young and normally have fertility needs, it has also been advocated that the therapeutic application of MBOT should be administered to treat IECA. However, there are currently no large-scale series of studies conducted for patients with IECA. Therefore, the scope of surgery, the need for postoperative chemotherapy, and the preservation of reproductive function in IECA patients have not yet been determined. The prognosis for borderline ovarian tumors (BOTs) is relatively favorable, with a 5-year survival rate of 90% or higher [10], while the prognosis for OC is more unfavorable, with a potentially high mortality rate among gynecologic malignancies and a 5-year survival rate of only 5–10% for advanced stage OC [11]. Yet younger patients with OC generally present at an earlier stage and have relatively better prognosis. Several studies have already demonstrated that adnexectomy is the preferable option in FSS regardless of BOTs and early-stage OC: patients who underwent adnexectomy had a postoperative recurrence-free interval (RFI) and cancer-specific survival that were not significantly different from those who underwent radical surgery, and generally possessed a better reproductive outcome [12–14]. It is necessary to clarify that although patients may remain capable of conceiving naturally after receiving FSS [15], ovarian reserve inevitably decreases, which has a negative consequence on fertility. Several cases have been reported of patients with BOT or OC applying ovulation induction, followed by oocyte cryopreservation, with subsequent embryo transfer resulting in a successful delivery, demonstrating that this technique is a promising alternative for fertility preservation in patients with ovarian tumors [16–18]. In this case, the patient failed to perform fertility preservation promptly after the initial completion of FSS, thus a favorable therapeutic opportunity was missed. Partly due to the economic reasons of the patient, but more importantly due to the lack of awareness about fertility preservation in such patients. It is very noteworthy that MBOT seems to be more prone to recurrence of invasive adenocarcinoma compared to serous BOT [19]. Although the above condition did not occur in this case, the patient developed a mucinous cystadenoma in her remaining ovary 9 years later, which suggests that we should pay attention to the possibility of recurrence or progression of MBOT with ICEA after conservative treatment, and that fertility preservation after the first FSS may result in a better reproductive outcome if it is performed, an aspect which we believe is a very significant pedagogical implication of this case. Fertility preservation were also didn’t performed before the second FSS, mainly due to the high-risk histologic factor IECA present in the first tumor and her relatively large cyst, with the possibility of puncturing the ovarian lesion during oocyte retrieval. It is worth emphasizing that in patients with recurrent BOT, if the first tumor is not accompanied by histological high-risk factors and the second treatment does not present any manifestation of clinically or radiologically malignant lesions [19], fertility preservation prior to the second FSS is feasible without affecting the overall prognosis [20], but it requires an experienced surgeon to perform the oocyte retrieval in order to prevent spreading of pathologic cells. After this patient underwent FSS again, there was a sudden drop in AMH and anterior follicle count (AFC), indicating a significant decline in ovarian function, but the timely application of ART on this occasion ultimately led to the patient’s successful conception, which suggests that the timeliness of applying ART after FSS in patients with bilateral ovarian tumors is extremely important.

EC is a common gynecologic cancer, and the age of onset in EC has been trending younger in recent years, with about 5% of women under the age of 40 at diagnosis and have not completed childbirth [21]. The standard treatment for endometrial cancer is hysterectomy and bilateral salpingo-oophorectomy, and patients who received that procedure have a favorable prognosis but are clearly deprived of their fertility [22]. Currently, EC patients can achieve a complete remission rate of more than 70% with the application of fertility-preserving treatment, but there remains a high risk of recurrence [23]. To date, a number of studies have reported that ART markedly improves patient pregnancy outcomes relative to spontaneous pregnancy and does not contribute to recurrence rates [24]. Since FFS for EC is not a standard surgical procedure and requires assessment every 3–6 months with a shorter duration for assisted conception, IVF-ET is presently more commonly used to assist conception in EC patients in clinical practice. The shorter the time interval between reaching CR and performing IVF, the greater the chance of successful birth in EC patients, so the timeliness of IVF in EC patients is also critical [25]. The patient in this case was only 36 years old and had a very strong need for reproduction with favorable postoperative pathology, which was consistent with the indication for fertility preservation, and was therefore treated conservatively and received IVF immediately afterwards.

When confronted with a dual primary cancer patient, it is essential to consider seriously the characteristics of each tumor before selecting an ovulation induction protocol. Fertility-preserving treatments in EC involve the application of progestin. Progestin has a protective effect on the endometrium by counteracting the effects of estrogen and promoting the transformation of the endometrium from the proliferative phase to the secretory phase. In addition, progestin also inhibits the luteinizing hormone (LH) surge and prevents premature ovulation [26]. Utilizing the principles mentioned above, the PPOS protocol has been clinically developed. The advantages of the PPOS protocol include antagonistic effects with estrogen-dependent disease and a certain degree of protection of the endometrium in patients with EC. The disadvantage is the unavailability of the fresh embryo transfer during the oocyte retrieval cycle, prolonging the duration of pregnancy. Chen et al. demonstrated that the PPOS regimen was effective in enhancing ovulation induction outcomes in EC patients without increasing the risk of recurrence [27]. However, in the present case, follicular dysplasia and low estrogen levels occurred during the treatment cycle with the application of the PPOS regimen, probably due to a prolonged application of MPA resulting in an overly intense direct suppression of GnRH in the hypothalamus by progesterone, which resulted in low gonadotropin (Gn) levels and failure of follicular growth, ultimately leading to the cancellation of this treatment cycle.

The GnRH antagonist protocol is another widely applied protocol in recent years, which has a relatively short treatment duration. GnRH antagonist is able to rapidly bind GnRH receptors and avoid inhibitory effects on the pituitary gland, while the amount of Gn applied during the ovulation induction cycle is relatively low, which may protect the ovaries comparatively and may also reduce the risk of recurrence of estrogen-sensitive cancers to some extent. Based on the advantages of the GnRH antagonist regimen described above, combined with the fact that in this case the patient had only one remaining ovary, which had declined in ovarian reserve as a result of receiving FSS, and was at risk of recurrence of IECA and a combination of EC. Therefore, the GnRH antagonist regimen was applied in the second treatment cycle and resulted in one top-quality embryo and one non-top-quality embryo, suggesting that the GnRH antagonist regimen is applicable in this type of patient, but the quality of the embryos and the success rate of subsequent pregnancies remain to be further investigated.

LE is a synthetic non-steroidal, highly selective aromatase inhibitor that competitively inhibits aromatase activity in vivo, blocks the conversion of androgens to estrogens, thereby reducing estrogen levels and inducing ovulation through both central and peripheral association pathways [28]. The mild ovarian stimulation regimen consisting of LE and Gn allows for peak estrogen levels within the ovulation-inducing cycle to be approximated to the natural cycle, without reducing the number of oocytes obtained and with an elevated live birth rate. Additionally, Marchetti et al. have indicated that LE can extend the RFI in OC patients and might be administered as maintenance therapy [29]. In this case, two top-quality embryos were retrieved after the application of a mild stimulation regimen containing LE, which suggests that letrozole has a nonnegligible advantage in inducing ovulation in patients with dual primary ovarian and endometrial cancer, but whether it reduces the recurrence risk needs to be determined in further prospective studies.

In addition to the need for careful thought when inducing ovulation in patients with dual primary gynecologic tumors, maternal and neonatal complications are also highly noteworthy. Placental disorders including placenta previa, placenta accreta, and others are a common complication of pregnancy, and IVF has been proven to be a significant risk factor for it [30]. Although the risk of placental disease was not elevated in OC patients who received FSS, a history of multiple intrauterine operations and application of MPA in EC patients treated conservatively increased such risk [31–33]. Placental disorders not only increase the risk of postpartum hemorrhage and puerperal infections leading to a higher maternal mortality rate, but also increase the risk of iatrogenic preterm birth (IPTB) and affect neonatal outcomes. A recent meta-analysis showed that the risk of IPTB in IVF/ICSI pregnancies is over twofold compared with spontaneous pregnancies, and the etiology is mostly related to placental dysfunction or abnormalities [34]. A history of dual primary tumors undoubtedly increases the risk of IPTB in addition to IVF and warrants more attention from physicians. Besides, fetal weight abnormalities are another concern. Several studies have now demonstrated a higher likelihood of large for gestational age (LGA) and a lower risk of small for gestational age (SGA) in frozen ET births compared to fresh ET [35]. Currently, there are two explanations for this observation, one of which is that epigenetic modification occurs during the freezing and thawing of the embryo, which in turn affects the changes in fetal growth potential [36]. Another explanation is that the endometrium and uterine cavity in fresh ET are considered to be in a supraphysiological condition as a result of high estradiol due to the ovulation induction [36], and this hypothesis explains to some extent the eventual absence of SGA in our case: the peak estradiol of this patient did not reach supraphysiologic level. In addition, confounders such as pre-pregnancy BMI, weight gain during pregnancy, and others were not controlled for in previous studies, which may also be a reason for the eventual occurrence of LGA in our case. The patient’s pre-pregnancy BMI was 25.4 kg/m^2^, which is not only a potential risk factor for EC but equally for LGA. To summarize, because of an extremely challenging fertility preservation and the high risk of maternal and neonatal complications women with dual primary gynecologic tumors, the entire procedure should be performed only in tertiary care centers with extensive ART experience in oncology patients and a multidisciplinary team to ensure the safety of both maternal and infant.

The patient still had a strong need for fertility, which made subsequent treatment a dilemma. Considering her medical history of EC and MBOT with IECA, we believe that the first step that needs to be performed is to determine if the patient’s tumors has recurred or progressed. If not, an immediate MDT is required to assess whether her scarred uterus and tumor history are suitable for another pregnancy, and then either determine the patient’s appropriate interpregnancy interval or persuade her to undergo a standard surgical procedure.

In conclusion, for primary endometrial and ovarian cancer patients with fertility needs, the most crucial point in the process of treatment is not the novelty, but the doctors need to comprehensively consider the specific conditions of the patients (such as tumor type, stage, etc.) and then formulate the most appropriate oncological treatment plan, perform fertility preservation timely. Ovulation induction protocols should be selected individually and prudently on the premise of not increasing the risk of recurrence to induce the maximum number of oocytes possible for patients with the aim of ameliorating reproductive outcomes. Moreover, due to the result of the history of multiple intrauterine operations, great attention needs to be paid to the risk of postpartum hemorrhage due to placental factors, which needs to be proactively prevented and managed.

## Data Availability

All data generated or analyzed during this case are contained in the article.

## Abbreviations

AFC: Antral follicle count
AMH: Anti-Müllerian hormone
ART: Assisted Reproductive Techniques
BOTs: Borderline ovarian tumors
CR: Complete response
EC: Endometrial cancer
FSS: Fertility-sparing surgery
Gn: Gonadotropin
GnRH: Gonadotropin releasing hormone
hCG: Human chorionic gonadotropin
hMG: Human menopausal gonadotropin
IECA: Intraepithelial carcinoma
IPTB: Iatrogenic preterm birth
IVF-ET: In vitro fertilization-embryo transfer
LE: Letrozole
LGA: Large for gestational age
LH: Luteinizing hormone
MBOT: Mucinous borderline ovarian tumor
MC: Menstruation cycle day
MDT: Multi-disciplinary treatment
MPA: Medroxyprogesterone acetate
MRI: Magnetic resonance imaging
OC: Ovarian cancer
PPOS: Progestin-primed ovarian stimulation
RFI: Recurrence-free interval
SEER: Surveillance, Epidemiology, and End Results
SGA: Small for gestational age
UAE: Uterine artery embolization
